# Far-infrared protects vascular endothelial cells from advanced glycation end products-induced injury via PLZF-mediated autophagy in diabetic mice

**DOI:** 10.1038/srep40442

**Published:** 2017-01-10

**Authors:** Cheng-Hsien Chen, Tso-Hsiao Chen, Mei-Yi Wu, Tz-Chong Chou, Jia-Rung Chen, Meng-Jun Wei, San-Liang Lee, Li-Yu Hong, Cai-Mei Zheng, I-Jen Chiu, Yuh-Feng Lin, Ching-Min Hsu, Yung-Ho Hsu

**Affiliations:** 1 Department of Internal Medicine, School of Medicine, College of Medicine, Taipei Medical University, Taiwan; 2 Division of Nephrology, Department of Internal Medicine, Shuang Ho Hospital, Taipei Medical University, Taiwan; 3 Division of Nephrology, Department of Internal Medicine, Wan Fang Hospital, Taipei Medical University, Taiwan; 4 School of Medicine, National Defense Medical Center, Taiwan; 5 Institute of Medical Sciences, Tzu Chi University, Taiwan; 6 Department of Electronic and Computer Engineering, National Taiwan University of Science and Technology, Taiwan; 7 Graduate Institute of Applied Science and Technology, National Taiwan University of Science and Technology, Taiwan

## Abstract

The accumulation of advanced glycation end products (AGEs) in diabetic patients induces vascular endothelial injury. Promyelocytic leukemia zinc finger protein (PLZF) is a transcription factor that can be activated by low-temperature far-infrared (FIR) irradiation to exert beneficial effects on the vascular endothelium. In the present study, we investigated the influence of FIR-induced PLZF activation on AGE-induced endothelial injury both *in vitro* and *in vivo*. FIR irradiation inhibited AGE-induced apoptosis in human umbilical vein endothelial cells (HUVECs). PLZF activation increased the expression of phosphatidylinositol-3 kinases (PI3K), which are important kinases in the autophagic signaling pathway. FIR-induced PLZF activation led to autophagy in HUVEC, which was mediated through the upregulation of PI3K. Immunofluorescence staining showed that AGEs were engulfed by HUVECs and localized to lysosomes. FIR-induced autophagy promoted AGEs degradation in HUVECs. In nicotinamide/streptozotocin-induced diabetic mice, FIR therapy reduced serum AGEs and AGEs deposition at the vascular endothelium. FIR therapy also reduced diabetes-induced inflammatory markers in the vascular endothelium and improved vascular endothelial function. These protective effects of FIR therapy were not found in PLZF-knockout mice. Our data suggest that FIR-induced PLZF activation in vascular endothelial cells protects the vascular endothelium in diabetic mice from AGE-induced injury.

The rapid increase of diabetic patients causes diabetes mellitus (DM) to be a serious medical problem worldwide. Vascular complications are the most important cause of morbidity and mortality in diabetic patients, and mainly result from endothelial dysfunction and vascular inflammation^1^,^2^. DM-associated endothelial dysfunction further accelerates atherosclerosis and induces structural changes of arteries with tissue hypoperfusion and hypoxia^3^. In the late stage of diabetes, the patients often suffer from acute coronary syndromes, myocardial infarction with silent myocardial ischaemia, peripheral artery disease, and stroke^4^. There is no ideal therapy for DM-associated vascular complications so far. Diabetic patients urgently need new effective therapies for vascular complications to increase life expectancy and improve the quality of life.

Many studies have revealed that advanced glycation end products (AGEs) play a critical role in promoting diabetic vascular dysfunction and diabetes development^5^. Reducing sugars can modify proteins by Maillard reaction, which extremely accelerates under diabetes to form AGEs^6^. Recent findings suggest that N-ε-carboxymethyl-lysine (CML), a major AGE *in vivo*, is the key modulators for the development of nonproliferative retinopathy among type 2 diabetic patients^7^. Interaction between AGEs and their receptor (RAGE) increases both mitochondrial and nicotinamide adenine dinucleotide phosphate (NADPH) oxidase-dependent reactive oxygen species (ROS) generation^8^. Excessive ROS is cytotoxic and contributes to vascular inflammation, thrombosis and diabetic vascular complications^8^,^9^. Both RAGE-mediated ROS generation and high glucose promote apoptosis in endothelial cells by activating nuclear factor-kappa B (NF-κB), c-Jun NH2-terminal kinase and caspases^3^,^10^. NF-κB is a key molecule in regulating expression changes of inflammatory mediators such as inducible nitric oxide synthase (iNOS), intercellular adhesion molecule-1, and major histocompatibility complex class II^11^. Additionally, the activation of RAGE induce various pro-inflammatory mediators in endothelia cells, such as transforming growth factor beta 1 (TGF-β1), intercellular adhesion molecule-1 and monocyte chemotactic protein-1 (MCP-1)^12^,^13^. The pro-inflammatory mediators induce adhesion of monocyte/macrophage and lymphocytes to endothelial cells, which is also an early and central event in diabetic vascular dysfunction development^5^. Based on the studies described above, endothelial apoptosis and inflammation are major AGE-induced injury in diabetic vascular endothelium.

Promyelocytic leukaemia zinc finger protein (PLZF) is a nuclear transcription factor belonging to the BTB/POZ family^14^. It is recognized as an inducer of innate-like features in many different lymphocytes^15^. PLZF has also been directly linked to tumor suppression via its transcriptional repression of the *c-myc* oncogene^16^. Many cell types express PLZF to mediate various signaling, growth regulatory and diverse functions, such as homeostasis, neoplasia, and apoptosis^17^,^18^. Our recent study shows that low-temperature far-infrared (FIR) induces PLZF nuclear translocation to increase PI3K expression in human umbilical vein endothelial cells (HUVECs)^19^. The PI3K family (classes I and III) has been shown to stimulate autophagy^20^. Therefore, FIR-induced PLZF nuclear translocation has potential to induce autophagy in vascular endothelial cells.

Autophagy is a process involving degradation of long-lived proteins, which consists of the formation of autophagosomes, the fusion of autophagosomes and lysosomes, the degradation of autophagic cargo by the lysosomal hydrolases, and the recycling of the products^21^,^22^. Two proteins, beclin-1 and microtubule-associated protein 1 light chain 3 (LC3), play important roles in the process of autophagy. Beclin-1 is an integral protein in the class III PI3K pathway and triggers autophagy^23^. The cytoplasmic form of LC3 (LC3-I) becomes membrane-associated (LC3-II) to form autophagosome during autophagy^24^. Many studies show a protective role of autophagy in AGE-induced injury in HUVECs^25^,^26^. Considering the possibility of PLZF-mediated autophagy, FIR-induced PLZF nuclear translocation may protect vascular endothelial cells from AGE-induced injury. This study tested the hypothesis *in vitro* and *in vivo*, and revealed its autophagy-associated mechanism.

## Results

### FIR irradiation protects HUVECs from AGE-induced apoptosis

**Figure 1:**
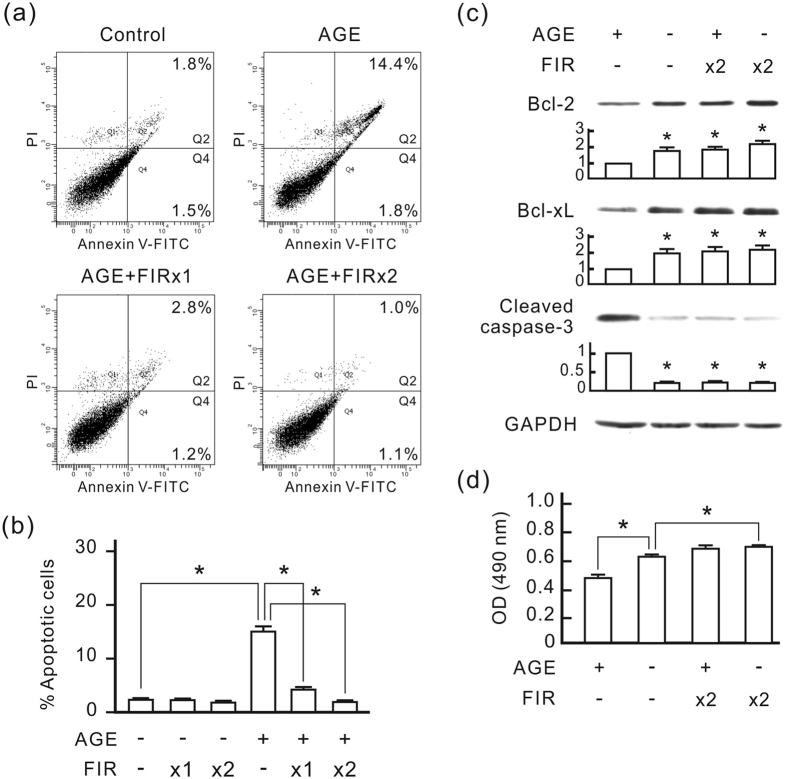
FIR irradiation inhibits AGE-induced apoptosis in HUVECs. HUVECs were treated with 250 μg/ml AGE-BSA and then cultured for 48 h. FIR irradiation was applied 30 min after the AGE-BSA treatment (x1), or 30 min and 24 h after the AGE-BSA treatment (x2). Each irradiation period is 30 min. Treated cells were stained with annexin V/PI and analyzed using flow cytometry. **(a)** Representative flow cytometric data. In each plot, the lower left quadrant represents viable cells, the lower right quadrant represents early apoptotic cells, and the upper right quadrant represents late apoptotic cells. **(b)** A bar chart form of the apoptotic data. Data are presented as the mean ± SD (n = 3). *P < 0.05. **(c)** The influence of FIR on AGE-induced apoptotic signals. GAPDH was detected as a loading control. The relative quantity of each band on Western blots is also presented in a bar chart. Data are presented as the mean ± SD (n = 3). *P < 0.0 vs. the AGE-treated cells. **(d)** The influence of FIR on cell viability. Cell viability results are presented as the absorbance of each sample at 490 nm. Data are presented as the mean ± SD (n = 3). *P < 0.05.

The treatment of 250 μg/ml AGE-bovine serum albumin (BSA) considerably induced apoptosis in HUVECs in 48 h as revealed by annexin V/PI double staining and flow cytometry (Fig. 1a and b). FIR irradiation dose-dependently reduced AGE-BSA-induced apoptosis. Western blot analysis revealed that AGE-BSA reduced Bcl-2 and Bcl-xL expression and increased cleaved caspases-3 in HUVECs (Fig. 1c). FIR irradiation considerably increased Bcl-2 and Bcl-xL expression, and decreased cleaved caspases-3 in AGE-BSA-treated cells. The cell viability assay revealed that AGE-BSA significantly reduced HUVEC viability, and FIR irradiation blocked the influence of AGE-BSA and mildly increased HUVEC viability (Fig. 1d).

### PLZF nuclear translocation is necessary for the anti-apoptotic effect of FIR

**Figure 2:**
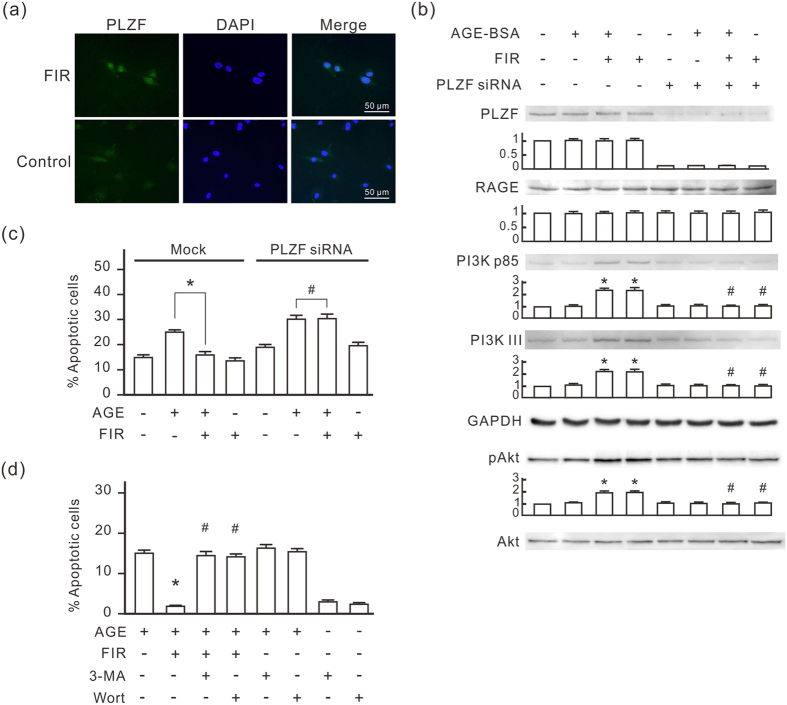
PLZF and PI3Ks are involved in FIR-induced anti-apoptotic effect in HUVECs. **(a)** FIR-induced nuclear translocation of PLZF. In FIR groups, HUVECs were exposed to FIR for 30 min, and then stained with PLZF antibody and DAPI for immunofluorescence staining. **(b)** The induction effect of FIR-induced PLZF activation on the expression of PI3Ks. HUVECs were transfected with PLZF siRNA or control siRNA, treated with or without AGE-BSA (250 μg/ml) for 30 min, and then exposed to FIR for 30 min. The relative quantity of each band on Western blots is also presented in a bar chart. Data are presented as the mean ± SD (n = 3). *P < 0.05 vs. the mock control cells. ^#^No significant difference from the PLZF siRNA-transfected cells. **(c)** The inhibitory effect of PLZF siRNA on the anti-apoptotic effect of FIR in AGE-BSA-treated cells. PLZF siRNA transfected HUVECs were treated with or without AGE-BSA for 48 h. FIR irradiation was applied 30 min and 24 h after the AGE-BSA treatment. Treated cells were stained with annexin V/PI and analyzed using flow cytometry. Data are presented as the mean ± SD (n = 3). *P < 0.05. ^#^No significant difference. **(d)** The inhibitory effect of 3-MA and wortmannin on the anti-apoptotic effect of FIR in AGE-BSA-treated cells. HUVECs were treated with 3-MA or wortmannin for 30 min, and then treated with AGE-BSA and FIR irradiation as described above. Treated cells were stained with annexin V/PI and analyzed using flow cytometry. Data are presented as the mean ± SD (n = 3). *P < 0.05 vs. the AGE-treated cells. ^#^No significant difference from the AGE-treated cells.

To confirm the influence of FIR irradiation on PLZF activation, we monitored the distribution of PLZF in HUVECs by immunofluorescence staining. As shown in Fig. 2a, FIR irradiation induced the translocation of PLZF to nuclei in HUVECs. Western blots revealed that FIR irradiation induced PI3K p85 and PI3K III expression, which was not influenced by AGE-BSA treatment (Fig. 2b). The expression of phospho-Akt was also increased by FIR irradiation, which indicated that total activity of PI3K increased in FIR-treated cells. Neither FIR nor AGE-BSA influenced the expression of RAGE. We used PLZF siRNA transfection to block PLZF expression in HUVECs, and then found that FIR irradiation failed to elevate the expression of PI3K p85, PI3K III, and phospho-Akt (Fig. 2b). PLZF siRNA transfection blocked the inhibitory effect of FIR on AGE-BSA-induced apoptosis in HUVECs (Fig. 2c). Two inhibitors of PI3Ks, 3-methyladenine (3-MA) and wortmannin, also inhibited the anti-apoptotic effect of FIR in AGE-BSA-treated HUVECs (Fig. 2d). These results reveal that FIR-induced PLZF nuclear translocation upregulates PI3Ks to inhibit AGE-BSA-induced apoptosis in HUVECs.

### FIR-induced PLZF activation induces autophagy in HUVECs

**Figure 3:**
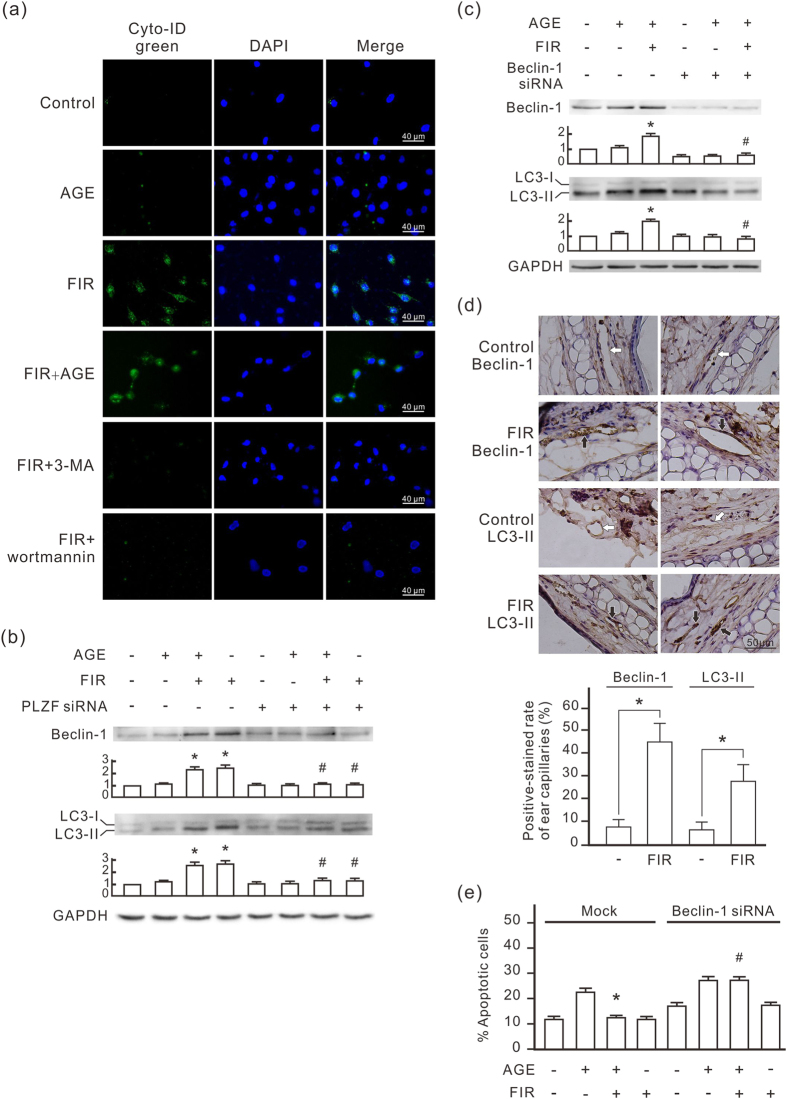
FIR induces autophagy in vascular endothelial cells via the PLZF signaling pathway. **(a)** FIR-induced autophagy in HUVECs. Cells were pretreated with 1 mM 3-methyladenine (3-MA) or 1 nM wortmannin for 30 min, treated with AGE-BSA (250 μg/ml) for 30 min, and then exposed to FIR for 30 min as indicated. Autophagy detection was performed by Cyto-ID Green staining. Scale bar = 40 μm. **(b)** The involvement of PLZF in the FIR-induced autophagy. HUVECs were transfected with PLZF siRNA or control siRNA. The relative quantity of beclin-1 and LC3-II bands on western blots is also presented in bar charts. Data are presented as the mean ± SD (n = 3). *P < 0.05 vs. the mock control cells. ^#^No significant difference from the PLZF siRNA-transfected cells. **(c)** The involvement of beclin-1 in the FIR-induced autophagic signaling pathway. HUVECs were transfected with beclin-1 siRNA or control siRNA, and then analyzed by Western blotting. Data are presented as the mean ± SD (n = 3). *P < 0.05 vs. the mock control cells. ^#^No significant difference from the beclin-1 siRNA-transfected cells. **(d)** FIR-induced beclin-1 and LC3-II *in vivo*. Mice were exposed to FIR for 30 min. The expression of beclin-1 and LC3-II in microvascular endothelium in ears was monitored by IHC. The white and black arrows indicate the negative- and positive-stained vascular endothelium respectively. The positive rate in ear microvessels are also presented as the mean ± SD (n = 6). *P < 0.05. Scale bar = 50 μm. **(e)** The influence of beclin-1 siRNA on the anti-apoptotic effect of FIR. HUVECs were transfected with beclin-1 siRNA or control siRNA, and then treated with AGE-BSA (250 μg/ml) for 48 h as indicated. Thirsty-min FIR irradiation was applied 30 min and 24 h after the AGE-BSA treatment. Treated cells were stained with annexin V/PI and analyzed using flow cytometry. Data are presented as the mean ± SD (n = 3). * *P* < 0.05 vs. the AGE-treated mock control cells. ^#^No significant difference from the beclin-1 siRNA-transfected cells treated with AGE.

Because PI3K classes I and III were involved in autophagy, we further evaluated the connection between FIR and autophagy using Cyto-ID Green that selectively labels autophagic vacuoles. As shown in Fig. 3a, FIR irradiation induced obvious autophagic vacuole accumulation in HUVECs. Autophagic vacuole accumulation appeared weak in the cells with AGE-BSA treatment alone. Both 3-methyladenine (3-MA) and wortmannin completely inhibited FIR-induced autophagic vacuole accumulation autophagic vacuole accumulation. Western blots revealed that FIR irradiation increased beclin-1 and LC3-II expression in HUVECs, which was blocked by PLZF siRNA transfection (Fig. 3b). AGE-BSA also increased beclin-1 and LC3-II expression in HUVECs but the effect was weaker than that of FIR irradiation. We used beclin-1 siRNA transfection to block beclin-1 expression in HUVECs, and then found that FIR irradiation failed to elevate LC3-II expression (Fig. 3c). FIR-induced autophagic signals were also confirmed *in vivo*. Vascular endothelium in the ears of FIR-irradiated mice expressed higher levels of beclin-1 and LC3 compared with that in control mice (Fig. 3d). Beclin-1 siRNA transfection also blocked the inhibitory effect of FIR on AGE-BSA-induced apoptosis in HUVECs (Fig. 3e). These results suggest that FIR-induced PLZF activation induces autophagy in vascular endothelial cells, which plays a critical role in the anti-apoptotic effect of FIR.

### PLZF-mediated autophagy promotes AGEs degradation in HUVECs

**Figure 4:**
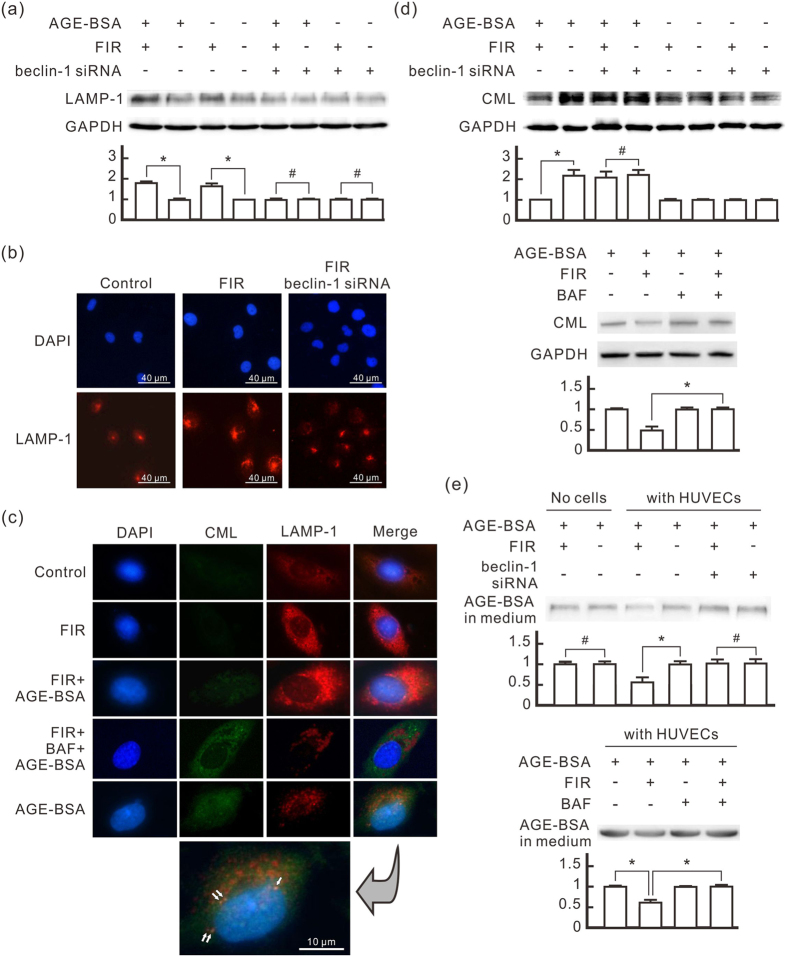
FIR-induced autophagy promotes AGE-BSA degradation in HUVECs. HUVECs were transfected with beclin-1 siRNA or pretreated with bafilomycin A1 (BAF, 25 nM) for 30 min, treated with AGE-BSA (250 μg/ml) for 30 min, and then exposed to FIR for 30 min as indicated. **(a)** The induction effect of FIR-induced autophagy on the lysosomal membrane protein LAMP-1 expression. The treated cells were cultured for 1 h after FIR irradiation. The relative quantity of LAMP-1 bands on Western blots is also presented in a bar chart. Data are presented as the mean ± SD (n = 3). **(b)** Immunofluorescence staining of LAMP-1 in HUVECs. The cells were stained with DAPI and anti-LAMP-1 antibody. Scale bar = 40 μm. **(c)** Co-localization of AGEs and lysosomes in HUVECs. The cells were stained with DAPI, anti-BSA and anti-LAMP-1 antibodies. The white arrows indicate the yellow spots, representing co-localization of AGEs and lysosomes. Scale bar = 10 μm. **(d)** The reducing effect of FIR-induced autophagy on intracellular AGEs accumulation in HUVECs. The treated cells were cultured for 24 h after FIR irradiation. Western blot analysis was used to detect intracellular CML. The relative quantity of CML bands is also presented in a bar chart. Data are presented as the mean ± SD (n = 3). **(e)** The reducing effect of FIR-induced autophagy on extracellular AGEs in HUVECs. We added 3 ml medium in each 6-cm plate with or without HUVECs, and then incubated it with AGE-BSA (100 μg/ml) and bafilomycin A1 (BAF, 25 nM) for 48 h as indicated. HUVECs were exposed to FIR at 30 min and 24 h after the AGE-BSA treatment. Each irradiation period is 30 min. Fifteen microliter medium from each sample was applied in western blot analysis with anti-BSA antibody. Data are presented as the mean ± SD (n = 3). *P < 0.05. ^#^No significant difference.

Lysosomes are involved in the degradation of autophagic cargo. To evaluate the influence of PLZF-mediated autophagy on lysosome formation, we monitored the expression of the Lysosomal-associated membrane protein 1 (LAMP-1) in HUVECs. As shown in Fig. 4a, FIR irradiation considerably increased LAMP-1 expression, whether HUVECs were treated with AGE-BSA or not. Beclin-1 siRNA transfection blocked FIR-induced LAMP-1 increase. Immuno-fluorescence staining also showed that FIR irradiation increased LAMP-1 expression and scatter in HUVECs but not in beclin-1 siRNA-transfected cells (Fig. 4b). With AGE-BSA treatment, we found co-localization of LAMP-1 and CML in the HUVECs with AGE-BSA treatment alone, but not in the cells with AGE-BSA and FIR treatments (Fig. 4c). Both FIR-induced LAMP-1 and co-localization of LAMP-1 and CML were inhibited by bafilomycin A1, an inhibitor for acidification and protein degradation in lysosomes (Fig. 4c). This result suggests that AGEs can be engulfed and accumulate in HUVECs, which is prevented by FIR irradiation. In Western blotting assays, we found that AGE-BSA treatment increased intracellular CML in HUVECs (Fig. 4d). FIR irradiation considerably decreased intracellular CML in AGE-BSA-treated cells. But this phenomenon was not found in beclin-1 siRNA-transfected cells. FIR didn’t influence the intracellular CML level in bafilomycin A1-treated HUVECs at all (Fig. 4d), indicating that the involvement of lysosomes is necessary for FIR-reduced intracellular AGEs. This result also revealed that FIR didn’t influence the engulfment of AGEs in AGE-BSA-treated cells. In quantitative analyses, FIR irradiation didn’t influence the quantity of AGE-BSA in culture medium without cells (Fig. 4e). With the existence of HUVECs, however, FIR irradiation decreased AGE-BSA in culture medium, which was blocked by beclin-1 siRNA transfection and bafilomycin A1. These results suggest that PLZF-mediated autophagy promotes AGEs degradation in HUVECs to reduce extracellular AGEs.

### FIR therapy reduces serum AGEs in diabetic mice

**Figure 5:**
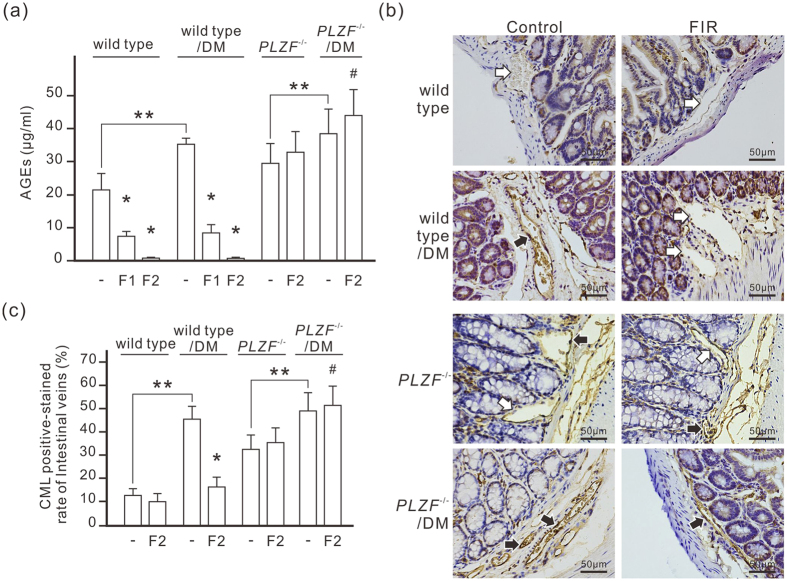
FIR-induced PLZF activation reduces serum AGEs in STZ-induced diabetic mice. **(a)** The reducing effect of FIR on serum AGEs in diabetic mice. Mice were exposed to daily FIR irradiation of 30 min for 1 week (F1) or 2 weeks (F2). DM, diabetic mice. Data are presented as the mean ± SD (n = 6 for wild type, n = 5 for *PLZF*^−/−^ mice). **(b)** The reducing effect of FIR on AGEs in vascular endothelium. Diabetic mice were exposed to daily FIR irradiation of 30 min for 2 weeks. The large intestines from each mouse were stained by immunohistochemistry with anti-CML antibody. The white and black arrows indicate the negative- and positive-stained endothelium of intestinal microvessels respectively. Scale bar = 50 μm. **(c)** A bar chart form of positive-stained rate of intestinal microvessles. Data are presented as the mean ± SD (n = 6 for wild type, n = 5 for *PLZF*^−/−^ mice). * *P* < 0.05 vs. the wild-type mice without FIR irradiation in the same group. ^#^No significant difference from the *PLZF*^−/−^/DM mice without FIR irradiation. **P < 0.05.

To further evaluate PLZF-mediated AGEs degradation *in vivo*, we treated nicotinamide-STZ induced type 2 diabetic mice with daily FIR irradiation of 30 min for 1 or 2 weeks. The oral glucose tolerance test (OGTT) revealed that diabetic mice had higher blood glucose levels during 135 min period compared to normal mice (Supplementary Figure S1). FIR therapy didn’t influence the oral glucose tolerance in diabetic mice. Monitoring serum AGEs by ELISA showed that the level of AGEs in diabetic mice was higher than in control mice (35.78 ± 1.46 μg/ml vs. 21.27 ± 6.17 μg/ml) (Fig. 5a). FIR therapy reduced the serum AGEs in both normal and diabetic mice in a dose-dependent manner (Fig. 5a). In *PLZF*^−/−^ mice, the basal level of serum AGEs (29.68 ± 3.75 μg/ml) was higher than in wild-type mice although the fasting serum glucose was similar to that in wild-type mice (99 ± 16 mg/dl vs. 98 ± 11 mg/dl). Diabetes further increased serum AGEs to 38.78 ± 8.38 μg/ml in *PLZF*^−/−^ mice. FIR therapy failed to reduce serum AGEs in *PLZF*^−/−^ mice, but slightly increased serum AGEs instead (Fig. 5a). IHC results showed that very little CML was detected in vascular endothelium in healthy mice, while obvious endothelial CML was found in diabetic mice (Fig. 5b,c). The endothelial CML in *PLZF*^−/−^ mice was also obvious and was further increased by diabetes. FIR therapy for 2 weeks considerably reduced endothelial CML in diabetic mice, but had no influence on *PLZF*^−/−^ mice (Fig. 5b,c). Co-immunostaining for CD31 (an endothelial marker) and CML also confirmed CML deposition at vascular endothelium (Supplementary Figure S2). These results suggest that FIR-induced PLZF activation reduces AGEs in serum and vascular endothelium.

### FIR therapy reduces endothelial apoptosis and recovers vascular endothelial function in diabetic mice

**Figure 6:**
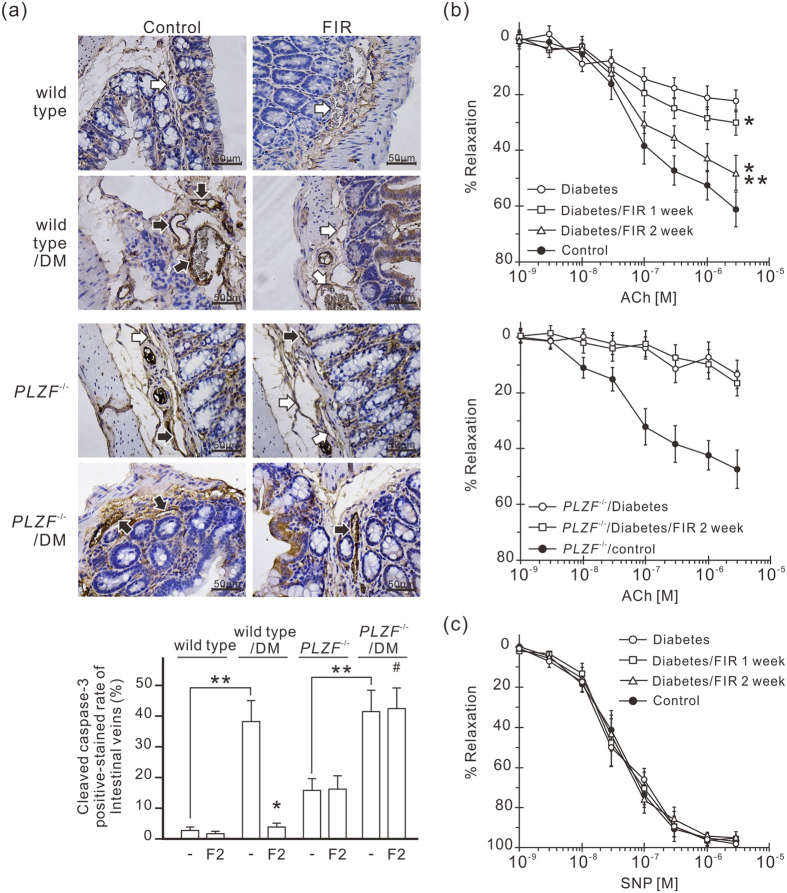
FIR-induced PLZF activation protects vascular endothelium in diabetic mice. **(a)** The inhibitory effect of FIR on the expression level of cleaved caspase-3 in the endothelium of intestinal microvessels. Mice were exposed to daily FIR irradiation of 30 min for 2 weeks. The intestines from each mouse were stained by immunohistochemistry with anti-cleaved caspase-3 antibody. The white and black arrows indicate the negative- and positive-stained endothelium of intestinal microvessels respectively. The staining results are also presented as a bar chart form of positive-stained rate of intestinal microvessels. DM, diabetic mice. *P < 0.05 vs. the DM wild-type mice without FIR irradiation. ^#^No significant difference from the *PLZF*^−/−^/DM mice without FIR irradiation. **P < 0.05. Scale bar = 50 μm. **(b)** Acetylcholine (ACh)-induced relaxation in aortic rings of diabetic mice. Diabetic wild-type and *PLZF*^−/−^ mice were exposed to daily FIR irradiation of 30 min for 2 weeks. **(c)** Sodium nitroprusside (SNP)-induced relaxation in aortic rings of diabetic mice. ACh- and SNP-induce relaxation of the thoracic aortic rings were measured and expressed as percent of precontraction. Each point of the curve comes from 5 independent experiments with thoracic aortic rings in each group (the mean ± SD). *P < 0.05 vs. DM wild-type mice without FIR irradiation. **P < 0.05 vs. DM wild-type mice with 1-week FIR irradiation.

FIR-induced AGEs reduction is supposed to reduce endothelial apoptosis and improve vascular function in diabetic mice. We monitored endothelial apoptosis in vascular endothelium by cleaved caspase-3 IHC staining, and found more cleaved caspase-3-positive stained endothelial cells in diabetic mice compared to in control mice (Fig. 6a). FIR therapy considerably decreased cleaved caspase-3 expression in vascular endothelium in diabetic mice. But the influence of FIR therapy on cleaved caspase-3 was unapparent in diabetic *PLZF*^−/−^ mice. This result suggests that FIR-induced PLZF activation inhibits diabetes-induced endothelial apoptosis. We also monitored acetylcholine-induced vessel relaxation to test the influence of FIR therapy on endothelial dysfunction in diabetic mice. Acetylcholine-elicited vessel relaxation was severely impaired in diabetic mice (Fig. 6b). FIR therapy considerably improved the relaxation in response to acetylcholine in diabetic mice, which was blocked by PLZF deficiency (Fig. 6b). The endothelium-independent NO donor sodium nitroprusside-induced relaxation was not significantly different in all groups (Fig. 6c). This result suggests that FIR-induced PLZF activation improves diabetes-induced vascular endothelial dysfunction.

### FIR therapy reduces endothelial inflammation in diabetic mice

**Figure 7:**
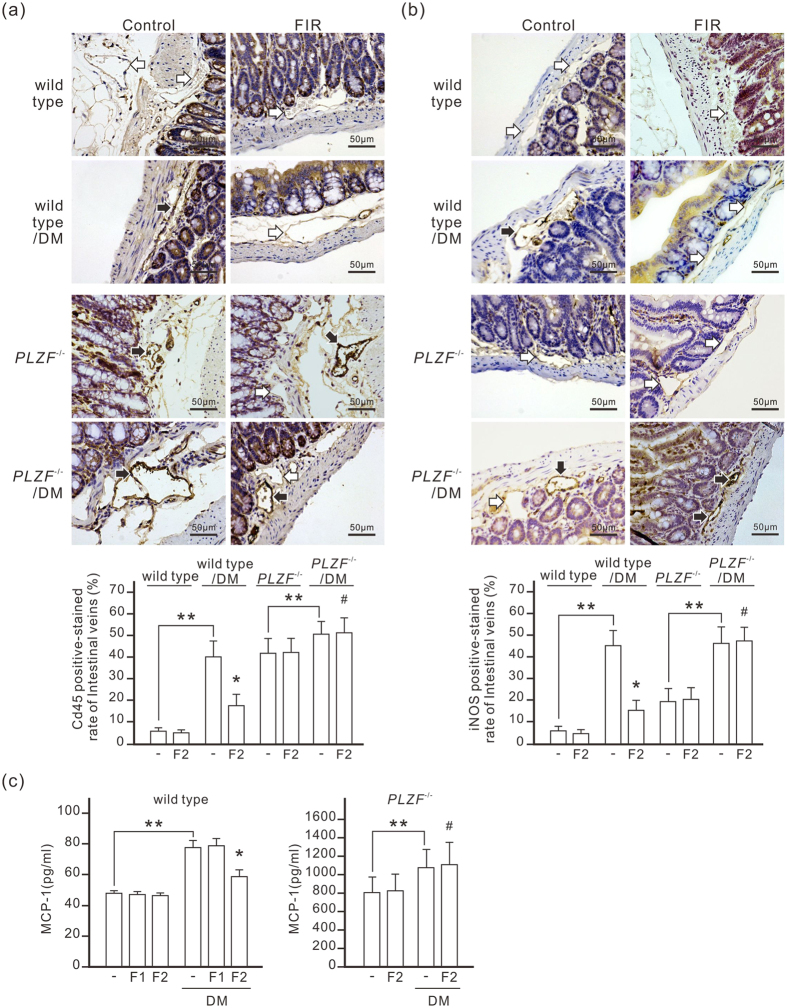
FIR-induced PLZF activation reduces vascular endothelial inflammation in diabetic mice. Mice were exposed to daily FIR irradiation of 30 min for 2 weeks (F2). The white and black arrows indicate the negative- and positive-stained endothelium of intestinal microvessels respectively. The staining results are also presented as a bar chart form of positive-stained rate of intestinal microvessels (the mean ± SD (n = 6 for wild type, n = 5 for *PLZF*^−/−^ mice)). DM, diabetic mice. *P < 0.05 vs. the DM wild-type mice without FIR irradiation. ^#^No significant difference from the *PLZF*^−/−^/DM mice without FIR irradiation. **P < 0.05. Scale bar = 50 μm. **(a)** The inhibitory effect of FIR on the expression level of CD45 in the endothelium of intestinal microvessels. The intestines from each mouse were stained by immunohistochemistry with anti-CD45 antibody. **(b)** The inhibitory effect of FIR on the expression level of iNOS in the endothelium of intestinal microvessels. **(c)** The reducing effect of FIR on serum MCP-1 levels in diabetic mice. Diabetic mice were exposed to daily FIR irradiation of 30 min for 1 (F1) or 2 weeks (F2). Serum MCP-1 was detected by ELISA.

The adhesion of leukocytes to endothelial cells is an early event in diabetic vascular dysfunction development. Immunochemistry staining revealed that diabetes considerably increased the expression level of CD45, a leukocyte common antigen, in vascular endothelium, suggesting leukocyte adhesion to endothelial cells increased in diabetic mice (Fig. 7a). Diabetes also considerably increased the expression level of iNOS, typically synthesized in response to inflammatory stimuli, in vascular endothelium (Fig. 7b). FIR therapy effectively reduced leukocyte-endothelial adhesion and iNOS expression in vascular endothelium of diabetic wild-type mice, but not in diabetic *PLZF*^−/−^ mice. The serum MCP-1 concentration in diabetic mice was higher than in control mice (Fig. 7c). FIR therapy considerably reduced serum MCP-1 in diabetic mice. In *PLZF*^−/−^ mice, the basal level of serum MCP-1 was 16 times higher than in wild-type mice. Diabetes further increased serum MCP-1 in *PLZF*^−/−^ mice, which was not influenced by FIR therapy. These results suggest that FIR-induced PLZF activation considerably reduces endothelial inflammation in diabetic mice.

## Discussion

PLZF is a transcription factor involved in major developmental and biological processes^27^. However, the protective role of PLZF in DM-associated endothelial dysfunction has not been documented. In the present study, we found that PLZF nuclear translocation reduced AGE-induced injury in vascular endothelial cells *in vitro* and *in vivo*. In HUVECs, we confirmed that FIR irradiation induced nuclear translocation of PLZF (Fig. 2). FIR-induced PLZF activation inhibited AGE-induced apoptosis and induced the expression of phosphatidylinositol-3 kinases (PI3Ks) (Figs 1 and 2). FIR irradiation also induced autophagy in HUVECs, which was blocked by PLZF siRNA transfection (Fig. 3). Beclin-1 siRNA transfection blocked FIR-induced autophagy and anti-apoptotic effect (Fig. 3). AGE-BSA has been found to be ingested by microvascular endothelial cells, which could contribute to the pathogenesis of diabetic microangiopathy^28^. FIR-induced PLZF activation promoted AGE-BSA degradation in HUVECs through the involvement of autophagosomes and lysosomes (Fig. 4). Additionally, the lysosome inhibitor bafilomycin A1 also reduced FIR-induced autophagic signals in HUVECs (Supplementary Figure S3). These results suggest that FIR-induced PLZF activation induces autophagy to promote AGEs degradation, and then reduces AGE-induced apoptosis in HUVECs. We also found that FIR therapy induced the expression of autophagic markers in vascular endothelium of mice (Fig. 3). Similar to the *in vitro* data, FIR therapy reduced serum AGEs in a dose-dependent manner in diabetic mice (Fig. 5a). FIR therapy also reduced endothelial AGEs, iNOS expression and leukocyte infiltration in the intestinal vascular endothelium in diabetic mice (Figs 5, 6 and 7). Diabetes-induced serum MCP-1 was decreased by FIR therapy (Fig. 7c). Aorta ring relaxation analysis revealed that FIR therapy improved vascular endothelial function in diabetic mice (Fig. 6b). However, these protective effects of FIR therapy didn’t exist in *PLZF*^−/−^ mice. Our data suggest that PLZF activation is a potential therapeutic target for DM-associated vascular complications.

**Figure 8:**
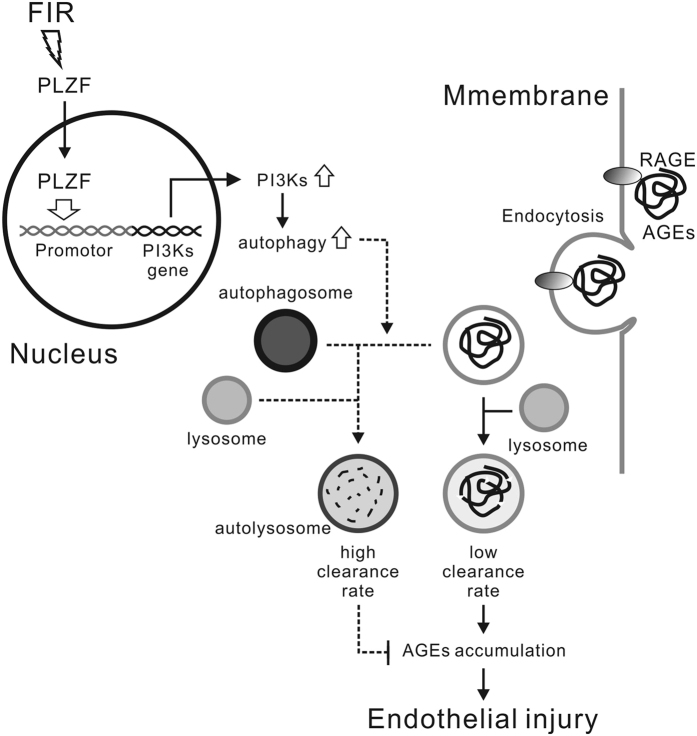
Schematic representation of the mechanism of FIR protecting vascular endothelial cells from AGEs-induced injury.

Some studies show that autophagy protects HUVECs from AGE-induced injury^25^,^26^. However, the detailed protective mechanism of autophagy is still unclear. Our study provides the first evidence that promoting AGEs degradation in vascular endothelial cells plays a role in the protective mechanism of autophagy. The clearance of serum AGEs used to be thought to be associated with macrophages and splenic reticuloendothelial cells^29^. Our results reveal that vascular endothelial cells are also involved in the clearance of serum AGEs. Recently, a diabetic study revealed that AGEs enter proximal tubule epithelial cells via macropinocytosis^30^. Macropinocytosis is a form of endocytosis, and mediates the engulfment of large volumes of extracellular fluid to form macropinosomes at the base of cell membrane ruffles^31^. Macropinocytosis may play an important role in AGEs engulfment and degradation in vascular endothelial cells. However, if AGEs degradation is not efficient, AGEs will accumulate in cells. AGEs accumulation has been proven to be harmful to cells^30^. Our results revealed that autophagy accelerated AGEs degradation in vascular endothelial cells and reduced extracellular AGEs (Fig. 4). The autophagic-lysosomal pathway is considered the main mechanism involved in removal of misfolded proteins and cell debris^32^. The engulfment of AGEs and autophagy let vascular endothelial cells have potential to decrease extracellular AGEs. In our data, FIR therapy reduced serum AGEs in both healthy and diabetic mice. The levels of serum AGEs in PLZF knockout control mice were similar to those in normal diabetic mice. These data suggest that PLZF-mediated autophagy in vascular endothelial cells plays an important role in the clearance of serum AGEs (Fig. 8).

Chronic hyperglycemia is a major feature of DM and induces protein glycation reactions leading to AGEs. AGEs are thought to be the major causes of different diabetic complications^7^. AGEs promote both endothelial cell apoptosis and vascular inflammation that contribute to endothelial dysfunction. In our system, FIR-induced AGEs clearance blocks endothelial cell apoptosis in diabetic mice as expected. However, the levels of leukocyte adhesion to endothelium, iNOS in endothelial cells, and serum MCP-1 in FIR-treated diabetic mice were still higher than the basal level of control mice, although they were lower than those in non-treated diabetic mice (Fig. 7). These results suggest that AGEs are not the only trigger for vascular inflammation in diabetic mice. Recent studies have demonstrated that transient hyperglycemia leads to epigenetic changes in gene promoters resulting in persistent inflammatory responses in endothelial cells^33^. That is to say high glucose also plays a role in triggering vascular inflammation in diabetic mice. However, FIR-induced AGEs clearance still improved diabetes-induced vascular dysfunction and inflammation. AGEs reduction as a whole should be a feasible strategy to keep vascular health in diabetic patients.

Far infrared therapy has been reported to promote new vessel formation in STZ-induced diabetic mice and may be associated with improved quality of life in people with type II diabetes mellitus^34^,^35^. The mechanism of these beneficial effects is a puzzle so far. The biological effect of FIR was traditionally considered to be related to a thermal-related mechanism^36^. Although some studies showed that FIR therapy may possess a non-thermal effect, there is no enough direct evidence to support it^37^. Our previous study found that FIR irradiation induced PLZF nuclear translocation in HUVECs, which was independent of a thermal effect^19^. In the present study, we further found that FIR induced autophagy in endothelial cells via PLZF *in vitro* and *in vivo*. Some studies shows that autophagy plays a protective role in toxic protein–mediated neurodegeneration, liver disease, and cardiovascular disease^38^. Modulation of autophagy may become a potential treatment for these disease. FIR therapy is the only non-invasive modality to induce autophagy so far, and could also be utilized in the treatment of disease conditions in which autophagy serves as a protective pathway in other tissues. However, more studies are necessary to investigate the existence of FIR-induced autophagy in other cell types.

MCP-1 is one of the key chemokines that regulate migration and infiltration of monocytes/macrophages. In *PLZF*^−/−^ mice, the serum MCP-1 level is about 16 times higher than in wild-type mice, and also 10 times higher than in diabetic wild-type mice (Fig. 7c). Obviously, this result is impossible to result from AGEs because the levels of serum AGEs in *PLZF*^−/−^ mice and diabetic wild-type mice are similar (Fig. 5c). PLZF is shown to be a critical regulator of immune system development and function^27^. The dramatic increase of serum MCP-1 in *PLZF*^−/−^ mice may result from PLZF gene deficiency, although no report discusses the connection between MCP-1 and PLZF. However, diabetes further increased serum MCP-1 in *PLZF*^−/−^ mice, which was not influenced by FIR therapy (Fig. 7c). This result still points out the critical role of PLZF in the inhibitory effect of FIR on diabetes-induced inflammation.

FIR irradiation often possesses a thermal effect because thermal transmission always accompanies FIR emission. The longer irradiation time causes the higher temperature of the target environment. Our previous study revealed that the increase in FIR intensity or irradiation time gradually reduced the biological effect of FIR on PLZF nuclear translocation and its downstream signaling pathways^19^. FIR irradiation at 0.13 mW/cm^2^ for 30 min can express the maximum biological effect in cell studies^19^. In the present study, the FIR irradiation conditions were also applied in the animal studies. Furthermore, the treatment condition of AGE-BSA in cell studies is another concern. AGE-BSA is not a strong apoptosis inducer. For studing apoptosis, the AGE-BSA dose often ranges between 100 and 300 μg/ml, and the treatment time is 24 or 48 h^39^,^40^. The level of AGE-BSA-induced apoptosis is dose- and time-dependent. In this study, 250 μg/ml of AGE-BSA for 48 h was applied to induce obvious apoptosis in HUVECs.

In conclusion, FIR-induced PLZF nuclear translocation induces autophagy through PI3K pathway in vascular endothelial cells. The PLZF-mediated autophagy promotes AGEs degradation in HUVECs and the clearance of serum AGEs in diabetic mice, which further decreases AGE-induced endothelial injury *in vitro* and *in vivo*. Through the PLZF-associated pathway, FIR therapy could be a potential therapeutic modality to prevent vascular complications of diabetes.

## Methods

### Cell culture

HUVECs were purchased from Food Industry Research and Development Institute (Taiwan), and cultured in Medium 200 with low serum growth supplements purchased from Thermo Fisher Scientific (Waltham, MA, USA). They were grown until the monolayer became confluent. AGE-BSA used in cell treatment was purchased from BioVision (Milpitas, CA, USA). All other chemicals of reagent grade were obtained from Sigma-Aldrich (St. Louis, MO, USA).

### FIR exposure

Cells were exposed to FIR emitted from a ceramic FIR generator, WS TY101 FIR emitter (WS Far Infrared Medical Technology, Taipei, Taiwan) with the effective energy intensity of 0.13 mW/cm^2^. The control groups were covered with aluminum foil. The whole set was maintained in 37 °C and 5% CO_2_ culture condition for 30 min. For mouse experiments, the whole mouse was exposed to FIR at 0.13 mW/cm^2^ for 30 min per day. The control mice were exposed to lamps and shaded with aluminum foil from light.

### Apoptosis detection

Fluorescein isothiocyanate (FITC)-annexin V/propidium iodide (PI) double staining was used to detect apoptosis induced by AGE-BSA treatment. Treated HUVECs were harvested and washed twice with ice-cold PBS. Specific binding of FITC-annexin V and staining with PI were performed using an apoptosis detection kit (BD Biosciences, San Diego, CA, USA), according to the manufacturer’s instructions. The cells were then analyzed using flow cytometry.

### Cell viability assay

HUVECs (10^4^ cells/well) were cultured in a 96-well microtiter plate in a final volume of 200 μl/well of culture medium. After the treatment, cells were added 3-(4,5-dimethylthiazol-2-yl)-5-(3-carboxymethoxyphenyl)-2-(4- sulfophenyl)-2H-tetrazolium (MTS) and were incubated at 37 °C for 1 h. MTS assay observes the reduction of MTS to formazan in viable cells. Formazan absorbance was measured at 490 nm.

### Western blot analysis

Fifteen micrograms of total proteins were applied to each lane and analyzed by Western blotting. PLZF antibody purchased from Santa Cruz Biotechnology (Dallas, TX, USA) were diluted to 1:200 for the assay. CML antibody purchased from Cosmo Bio (Tokyo, Japan) were diluted to 1:200 for the assay. BSA antibody purchased from Abcam (Cambridge, MA, USA) were diluted to 1:2000 for the assay. Other antibodies were purchased from Cell Signaling Technology (Danvers, MA, USA) and diluted to 1:1000 for the assay. Data of protein bands on Western blots were also quantitated with QuantiScan software (Biosoft, Cambridge, UK).

### Short interfering (si)RNA transfection

The siRNAs used in this study and control siRNAs were purchased from Life Technologies (Grand Island, NY, USA). Cells were grown to 70% confluence, and transiently transfected siRNAs using the lipofectamine RNAiMAX reagent according to the manufacturer’s instructions (Life Technologies). The final concentration of siRNA for transfection was 10 nM.

### Cyto-ID green autophagy detection

Autophagy detection was performed by Cyto-ID Green Autophagy Kit (Enzo, Farmingdale, NY, USA) followed the manufacturer’s instructions. The results were observed by fluorescence microscopy.

### Immunofluorescence staining

Cells were fixed with anhydrous methanol at −20 °C for 10 min and blocked in 3% normal serum for 1 h. The cells were incubated with primary anti-PLZF (Santa Cruz Biotechnology, Dallas, TX, USA) at a working dilution of 1:50, or anti-LAMP-1 (Cell Signaling Technology) and anti-CML (Cosmo Bio) at a working dilution of 1:200, at room temperature for 1 h. The cells were then incubated for 1 h with diluted secondary antibodies of different color in the dark at room temperature. Cells were counterstained with 4’,6-diamidino-2-phenylindole (DAPI) in mounting medium (Blossom Biotechnologies, Taiwan) to demonstrate the presence of nuclei. Stained cells were examined under an Invitrogen EVOS microscope (Thermo Fisher Scientific, Waltham, MA, USA).

### STZ-induced diabetic mice

All animal experiments were approved by the Taipei Medical University Committee of Experimental Animal Care and Use (approval No. LAC-2014-0291), and performed in accordance with relevant guidelines and regulations. Male 8-week-old C57BL/6 J mice were obtained from Lasco Technology (Taiwan). PLZF knock-out (*PLZF*^−/−^) mice arose on the strain C57BL/6 J^41^, and were kindly gifts from Prof. Yung-Hao Ching (Department of Molecular Biology and Human Genetics, Tzu Chi University, Taiwan). Homozygous mutants were crossed to C3HeB/FeJ to generate F1 heterozygotes. The experimental mice (n = 6 per group for wild type, n = 5 per group for *PLZF*^−/−^ mice) were intraperitoneally injected 150 mg/kg nicotinamide (Sigma), then injected 75 mg/kg streptozotocin (STZ, Sigma, solved in 0.1 M citrate buffer saline) on daily basis for 5 days. After 2 weeks, the second round of nicotinamide-STZ injection was carried out if the blood glucose level remains low (under 300 mg/dl). Dose of STZ in the second round of injection was reduced to 50 mg/kg. Administration of nicotinamide before the streptozotocin injection to the mice protects β-cells from STZ-induced severe cytotoxic damages, leading to chronic DM by producing moderate hyperglycemia^42^,^43^. In the present study, the plasma insulin levels in diabetic mice were much higher than those in normal mice, but did not disappear completely (Supplementary Table S1). Some type 2 diabetes-induced changes were shown to develop in this model^44^. After FIR irradiation, ears were cut for detecting beclin-1 and LC3 in microvascular endothelium. Large intestines were harvested by laparotomy for detecting microvascular endothelial injury. For histological analysis, we fixed the harvested ears and intestines in 10% formalin and embedded in paraffin.

### Aortic ring relaxation assay

Thoracic aorta from mice were cut into rings of 3–4 mm width. Isometric tension was measured in aortic rings as described^45^. In brief, tension changes in the aortic ring were measured by using an isometric transducer (FT03C; Grass, West Warwick, RI, USA) and were recorded on a Grass polygraph. A tension of 1 g was applied and the rings were equilibrated for 60 min. Oxygenated Krebs-Henseleit solution was provided at 15 min intervals. Rings were precontracted with norepinephrine (10^−6^ M), and then concentration-response curves to acetylcholine (10^−9^–10^−6^ M) and sodium nitroprusside (10^−9^–10^−6^ M) were obtained.

### Immunohistochemistry (IHC)

We made 4 μm-thick sections of formalin-fixed paraffin-embedded specimens for IHC. Slides were deparaffinized in xylene and rehydrated with 100% ethanol, 95% ethanol and water serially. We immersed slides into antigen-retrieval buffer (10 mM Tri-sodium citrate dihydrate, 0.05% Tween-20, pH 6.0) boiled via water bath for 30 minutes, and stained the slides using an UltraVision Quanto HRP Detection kit (Thermo Scientific, Rockford, IL, USA) with specific antibodies according to the manufacturer’s instructions. Antibodies of CD45, iNOS were from Abcam, Cleaved caspase-3 antibody was from Proteintech (Rosemont, IL, USA), LC3 antibody was from Cell Signaling Technology, and beclin-1 antibody was from Bioss (Woburn, Massachusetts, USA). CML antibody was from TransGenic (Tokyo, Japan). Microvascular endothelium in the tissue slides was identified by Cd31 staining.

### The detection of serum AGEs and MCP-1

The serum of experimental mice was detected using AGE Competitive ELISA kit (Cell Biolabs, San Diego, CA, USA) and Mouse MCP1 ELISA kit (eBioscience, San Diego, CA, USA) according to the manufacturer’s instructions.

### Statistical tests

Data were presented as mean ± standard deviation (S.D.). Statistical differences between two groups were determined using the Student’s t-test. One-way ANOVA was used in aortic ring acetylcholine-induce relaxation assays. The differences were considered significant if the *P* value was smaller than 0.05.

## Additional Information

**How to cite this article**: Chen, C.-H. *et al*. Far-infrared protects vascular endothelial cells from advanced glycation end products-induced injury via PLZF-mediated autophagy in diabetic mice. *Sci. Rep.* **7**, 40442; doi: 10.1038/srep40442 (2017).

**Publisher's note:** Springer Nature remains neutral with regard to jurisdictional claims in published maps and institutional affiliations.

## Supplementary Material

Supplementary Information
