# Supplementary material for: Far-infrared protects vascular endothelial cells from advanced glycation end products-induced injury via PLZF-mediated autophagy in diabetic mice
Source: Sci Rep. 2017 Jan 10;7:40442. doi: 10.1038/srep40442 (PMC5223182; doi:10.1038/srep40442)
Supplement: Supplementary Information [file srep40442-s1.pdf]

Far-infrared protects vascular endothelial cells from advanced glycation end products-induced injury via PLZF-mediated autophagy in diabetic mice

Cheng-Hsien Chen[1,2,3,4], Tso-Hsiao Chen[1,3], Mei-Yi Wu[1,2], Tz-Chong Chou[5], Jia-Rung Chen[2], Meng-Jun Wei[2], San-Liang Lee[6], Li-Yu Hong[2], Cai-Mei Zheng[2], I-Jen Chiu[2], Yuh-Feng Lin[1,2], Ching-Min Hsu[7], Yung-Ho Hsu[1,2,4*]

[1] Department of Internal Medicine, School of Medicine, College of Medicine, Taipei Medical University, Taiwan.

[2] Division of Nephrology, Department of Internal Medicine, Shuang Ho Hospital, Taipei Medical University, Taiwan.

[3] Division of Nephrology, Department of Internal Medicine, Wan Fang Hospital, Taipei Medical University, Taiwan.

[4] School of Medicine, National Defense Medical Center, Taiwan.

[5] Institute of Medical Sciences, Tzu Chi University, Taiwan.

[6] Department of Electronic and Computer Engineering, National Taiwan University of Science and Technology, Taiwan.

[7] Graduate Institute of Applied Science and Technology, National Taiwan University of Science and Technology, Taiwan.

* Corresponding author

Yung-Ho Hsu

E-mail: yhhsu@tmu.edu.tw, Tel: +886-2-22490088 ext. 8156

Supplementary Table S1. The plasma insulin levels of the experimental mice.

	Normal	DM
Wild-type	2.43 ± 0.15 μg/dl	0.16 ± 0.09 μg/dl
PLZF$^{-/-}$	2.39 ± 0.22 μg/dl	0.18 ± 0.11 μg/dl

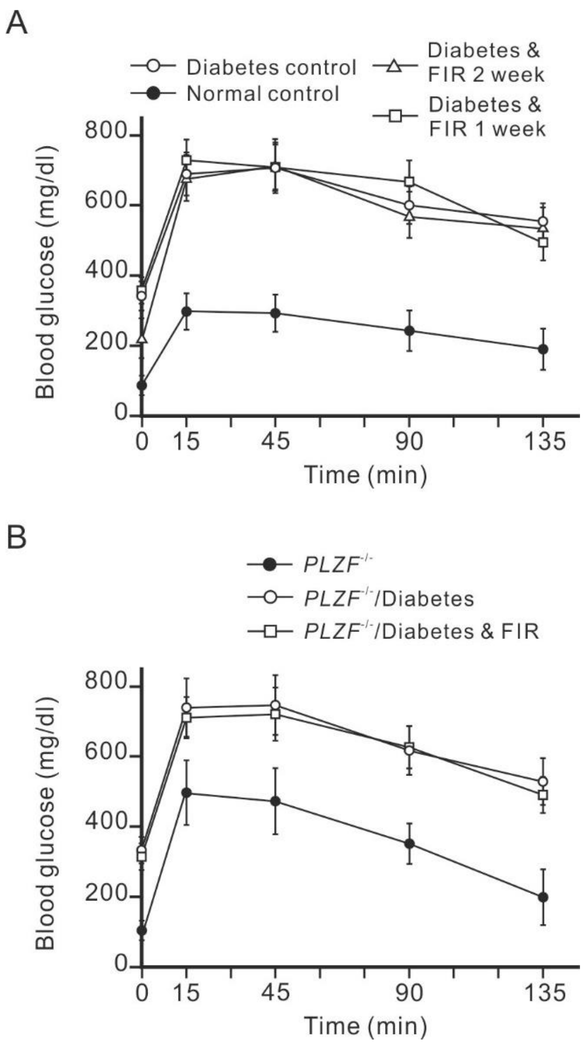

Supplementary Figure S1. Blood glucose concentrations during the course of the pre-operative 135 min OGTT in wild-type mice (A) and *PLZF*$^{-/-}$ mice (B). Diabetic mice were exposed to daily FIR irradiation of 30 min for 1 week or 2 weeks. OGTT was applied to confirm the diabetic syndrome in mice of diabetic groups. Before carrying out this experiment, mice were fasted for 12-16 hour. After measuring fasting blood glucose level, we fed mice with 2 g/kg glucose solution, and then measured blood glucose levels after 15, 45, 90, and 135 minutes.

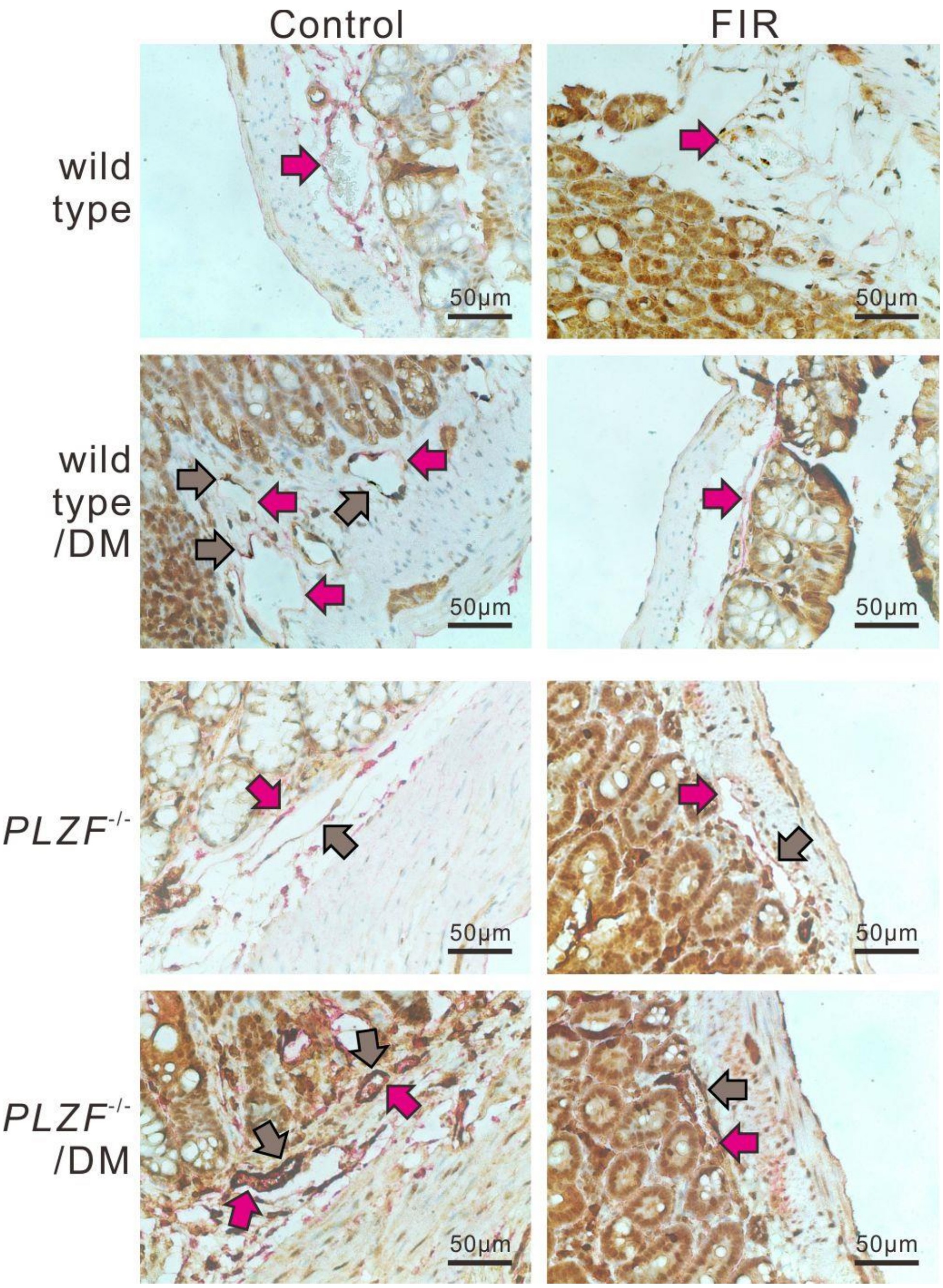

Supplementary Figure S2. Co-immunostaining for CD31 and CML. Diabetic mice were exposed to daily FIR irradiation of 30 min for 2 weeks. The large intestines from each mouse were stained by immunohistochemistry with ant-CD31 (Novus, Littleton, CO, USA, dilution 1:50) and anti-CML (dilution 1: 100) antibodies. Dual immunohistochemical stain was performed by the multivision polymer detection system (TL-012- MHRA, Thermo Fisher scientific, Waltham, MA, USA) using HRP-conjugated polymer (brown) for the anti-CML antibody and alkaline phosphatase-conjugated polymer (red) for the anti-CD31 antibody followed the manufacturer's instructions. The red and brown arrows indicate the CD31 and CML positive-stained regions respectively. Scale bar = 50 μm.

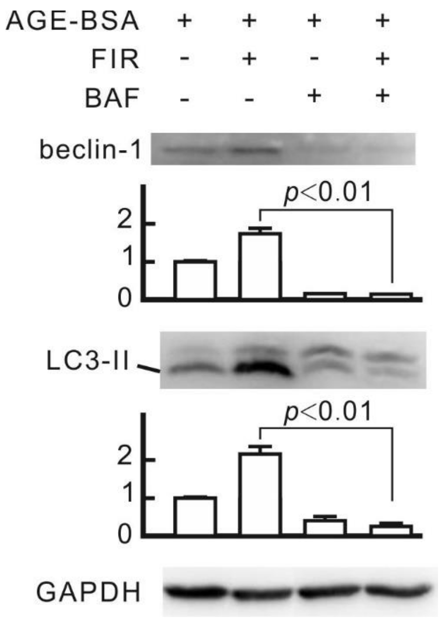

Supplementary Figure S3. The inhibitory effect of bafilomycin A1 on the FIR-induced autophagic signals. HUVECs were pretreated with bafilomycin A1 (25 nM, BAF) for 30 min, treated with AGE-BSA (250 μg/ml) for 30 min, and then exposed to FIR for 30 min as indicated. The cells were then cultured for 24 h. Western blot analysis was used to detect beclin-1 and LC3. GAPDH was detected as a loading control. The relative quantity of beclin-1 and LC3-II bands is also presented in bar charts. Data are presented as the mean ± SD (n = 3).

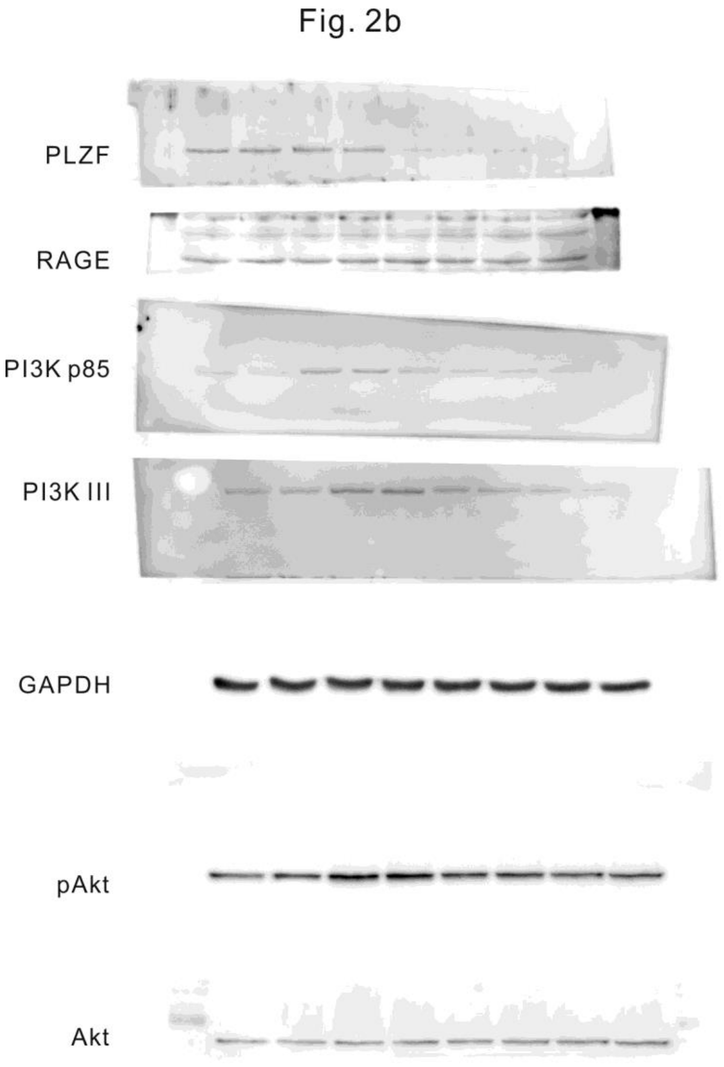

Supplementary Figure S4. The original blots in Figure 2.

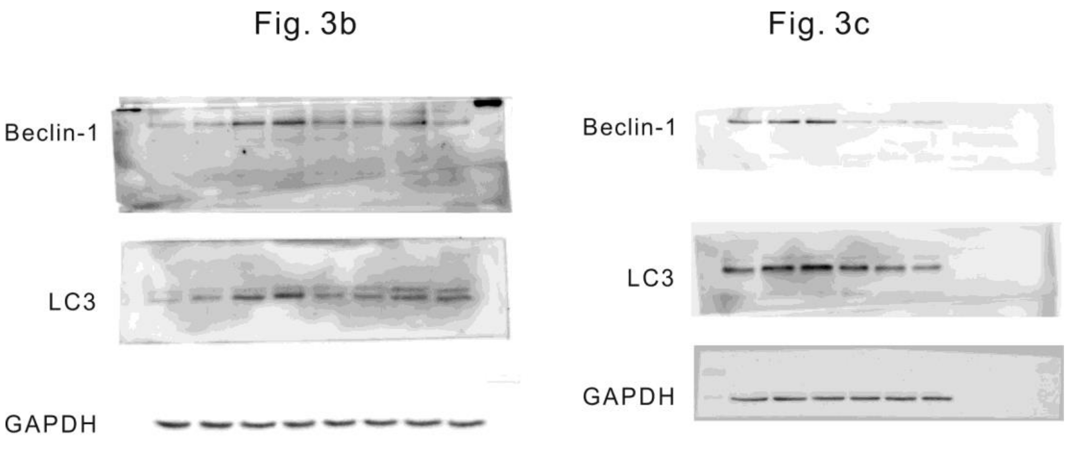

Supplementary Figure S5. The original blots in Figure 3.

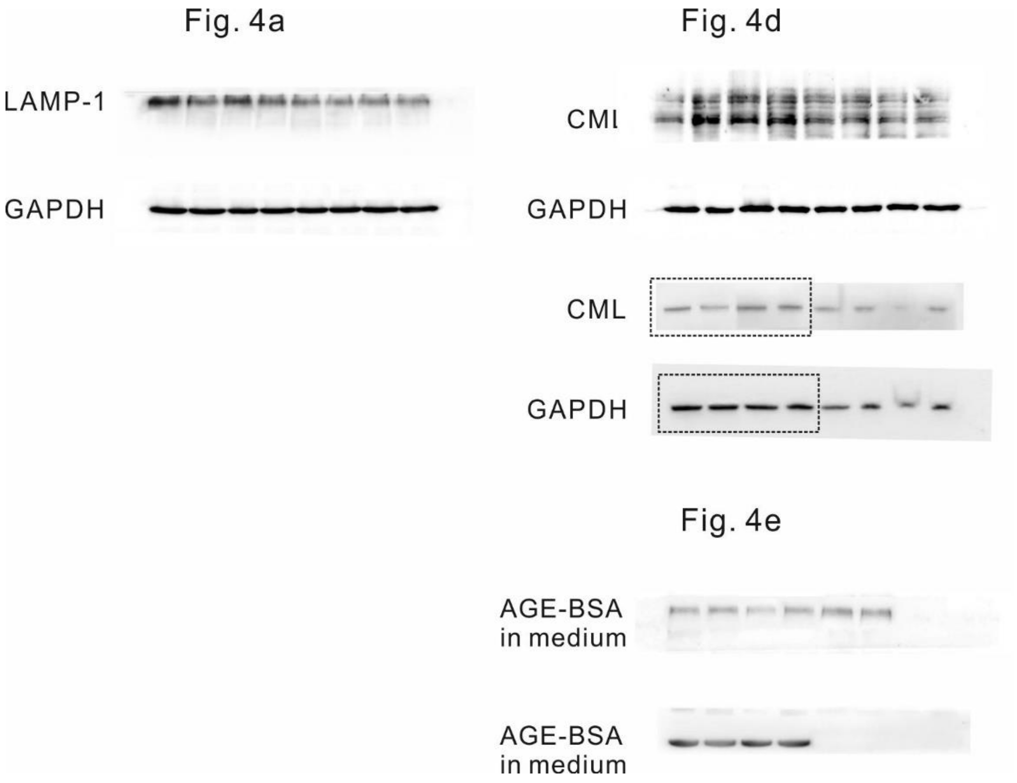

Supplementary Figure S6. The original blots in Figure 4.
